# Assessment and optimisation of normalisation methods for dual-colour antibody microarrays

**DOI:** 10.1186/1471-2105-11-556

**Published:** 2010-11-12

**Authors:** Martin Sill, Christoph Schröder, Jörg D Hoheisel, Axel Benner, Manuela Zucknick

**Affiliations:** 1 Division of Biostatistics, German Cancer Research Center, Im Neuenheimer Feld 280, Heidelberg, Germany; 2 Division of Functional Genome Analysis, German Cancer Research Center, Im Neuenheimer Feld 580, Heidelberg, Germany

## Abstract

**Background:**

Recent advances in antibody microarray technology have made it possible to measure the expression of hundreds of proteins simultaneously in a competitive dual-colour approach similar to dual-colour gene expression microarrays. Thus, the established normalisation methods for gene expression microarrays, e.g. loess regression, can in principle be applied to protein microarrays. However, the typical assumptions of such normalisation methods might be violated due to a bias in the selection of the proteins to be measured. Due to high costs and limited availability of high quality antibodies, the current arrays usually focus on a high proportion of regulated targets. Housekeeping features could be used to circumvent this problem, but they are typically underrepresented on protein arrays. Therefore, it might be beneficial to select invariant features among the features already represented on available arrays for normalisation by a dedicated selection algorithm.

**Results:**

We compare the performance of several normalisation methods that have been established for dual-colour gene expression microarrays. The focus is on an invariant selection algorithm, for which effective improvements are proposed. In a simulation study the performances of the different normalisation methods are compared with respect to their impact on the ability to correctly detect differentially expressed features. Furthermore, we apply the different normalisation methods to a pancreatic cancer data set to assess the impact on the classification power.

**Conclusions:**

The simulation study and the data application demonstrate the superior performance of the improved invariant selection algorithms in comparison to other normalisation methods, especially in situations where the assumptions of the usual global loess normalisation are violated.

## Background

While gene expression microarrays are now a standard tool in biological and medical research, microarray technologies for measuring protein expression are still in development. Antibody microarrays represent a technology that has potential for the screening of hundreds of protein expressions in parallel on large sample sets from minute sample volumes [1-3]. By specific antibodies immobilised on the microarray proteins are captured from complex protein samples which can be derived for example from blood, urine or tissue. In a so-called sandwich approach the captured proteins are then detected by a second set of antibodies specific for all target proteins. An alternative approach is based on a direct labelling of the protein samples and necessitates only a single capture antibody specific for each target protein. Thereby, it facilitates an easier scale-up to high content arrays of several hundreds to thousands of target proteins [4,5]. Additionally, such a setup enables a dual-colour layout, as it is commonly used in custom-made gene expression arrays. Herein, two samples are labelled by different fluorescent dyes (e.g. Cy3 and Cy5). In the subsequent incubation step they compete for the binding sites of the antibodies immobilised on the array. The signal intensities of the two dyes are measured for each spot by fluorecence image scanners and provide information on the relative abundance of the proteins under analysis in the respective samples. Dual-colour assay layouts proved their superior performance compared to single-colour assays in boutique antibody arrays with respect to reproducibility as well as discriminative power [6].

Due to the similar experimental setup, scanning and data acquisition infrastructure of cDNA microarrays can be utilised. Thereby, data are generated in a standard format, which facilitates the use of well-researched data handling, processing and statistical analysis tools of cDNA gene expression data, e.g. the open-source and open-development Bioconductor project [7].

For dual-colour cDNA array data the following steps are a vital part of the data pre-processing procedure to prevent technical artefacts from introducing unwanted systematic bias and variation (e.g. [7-9]). These steps are (i) filtering in order to remove failed and low-quality spots, (ii) background correction to correct for the general background fluorescence level due to non-specific binding, (iii) within-array normalisation to reduce variations between the two co-hybridised samples on each array and to remove dye-bias, and optionally, (iv) between-array normalisation to reduce variability between arrays. Since the dual-colour antibody array data are generated using a setup that is similar to the generation of dual-colour cDNA array data, the sources of bias and variation in the data are much the same and it seems reasonable to apply the same pre-processing steps as listed above.

However, antibody arrays have certain characteristic features which need to be taken into account specifically. First, it is much more difficult to quantify protein expression in a multiplex manner than for gene expression, due to the larger variability in the physico-chemical properties of proteins. Even after careful optimisation and tuning of the entire experimental design, the highly diverse electric charges and hydrophobicities of proteins which occur in complex samples usually lead to higher unspecific background binding than in DNA-microarrays. In addition, protein sizes as well as binding kinetics of the different antigen/antibody pairs vary much more than in DNA hybridisation experiments and the typical concentrations of proteins span a much broader range of magnitudes than for mRNAs. Consequently, it is much harder for protein arrays to design the array in such a way that the fluorescence intensities of all proteins are within the measurement limits of the scanner, increasing the likelihood of satiated data. Therefore, for a data analyst dealing with protein array data it is even more important to incorporate all sources of variation and bias properly in the data processing and modelling. Out of the data processing steps (i) to (iv) listed above, within-array normalisation is arguably the most important step with respect to the potential to help remove common sources of bias and is therefore the focus of this paper.

Due to splicing and post-translational modifications, the complexity of the human proteome is expected to range from the order of a hundred thousand to a million different protein molecules [10]. Apart from technical issues, there is a lack of well characterised antibodies against the majority of these proteins [10,11]. In addition the numbers of antibodies on current antibody microarrays are usually limited due to budget restriction and technical factors such as spatial limitations. Therefore, the typical application of antibody arrays is likely to be that of a boutique array, i.e. a moderately sized custom-built array targeting several hundreds to some thousands of proteins. Such arrays are usually designed to test the presence of a specific set of proteins which are known or suspected to be involved in a certain condition, for example in a certain type of cancer. Consequently, the data analyst has to deal with a considerable selection bias which might lead to a large proportion of proteins being differentially expressed between the tested conditions. Also, it is not unlikely that a majority of proteins is expressed in one direction, i.e. predominantly expressed in one of the two conditions that are being tested. Global loess normalisation techniques, which are very frequently used for normalisation of gene expression microarrays, assume that

(A) most probes are not differentially expressed and

(B) differential expression is symmetric, i.e. that over-expression and under-expression occur equally frequently

Both these assumptions might fail for boutique antibody arrays. Additionally, the number of non-regulated housekeeping controls represented on protein arrays will usually be small due to two facts. First, compared to transcriptional levels, there is only limited knowledge available on protein abundances in a variety of different tissues or body fluids in the presence or absence of a certain disease or treatment making it hard to select appropriate controls. Second, in antibody microarrays the costs per probe are about a factor of ten higher than for DNA-probes with unrestricted re-amplification possibilities. Therefore, usually the data analyst faces the problem, that only an inappropriate number of control features is available on the arrays, which could otherwise help to reliably estimate and remove systematic error. In this paper, we will concentrate on the crucial step of within-array data normalisation, which is important for removing systematic variations and biases within an individual array. The most important source of such variation arises from biases associated with the different fluorescent dyes ("dye-bias"). These biases can be dependent on the intensity levels, which are caused by scanning instruments, the label reaction as well as the chemical characteristics of the dyes them-selves. In addition, spatial variation across the array between the two dyes can occur. The intensity-dependent bias is typically addressed within the representation of the data in MA-plots [12], where the log_2 _expression ratios *M *= log_2 _(Cy5/Cy3) are plotted against the average log_2 _intensity values *A *= 0.5 × (log_2 _Cy5+log_2 _Cy3) (where Cy5 and Cy3 represent the filtered and background-corrected fluorescence intensity values for both dyes). After normalisation, the M-values should not depend on the A-values. Loess normalisation uses a robust local scatterplot smoothing method based on locally regressing the M-values on the A values and subsequent replacement by the regression residuals, which will have mean zero independently of the A-values. Due to nonlinear effects of the intensity-dependent bias, loess regression usually performs better than normalisation methods using linear regression or simply applying a constant shift in M-values independent of intensity values (e.g. median normalisation) [8,13]. However, just like these simpler methods loess normalisation relies on the assumptions (A) and (B) mentioned above. If these assumptions are not met, the M-values of truly differentially expressed proteins might be biased towards zero, potentially leading to a loss in detection and consequently to an artificial increase in false negative rates. Several methods have been proposed to adapt loess normalisation to situations where the assumptions (A) and (B) are not met by restricting the application of loess regression to a set of probes, that should not be differentially expressed. One example is the use of housekeeping probes [14,15], another is the inclusion of spike-in controls on the array, which can then be used for loess normalisation [15]. At the moment however, as it was the case in the early days of DNA microarrays, boutique protein arrays usually lack a sufficient number of such predefined control features [15-17]. Hence, we here pursue a third possibility, which is the use of algorithms to define sets of probes, which do not vary much in the dataset at hand with respect to their ranks in both dyes, i.e. so-called rank-invariant sets [8,18]. Previous simulations and data applications have indicated a superior performance of the rank-invariant method first proposed by Tseng et al. [8] compared to the global loess approach. We investigate this method in more detail and demonstrate an improved performance by adaptations of the algorithm determining the rank-invariant sets.

## Methods

### Rank-invariant selection algorithm (InvTseng)

In order to normalise boutique dual-colour microarrays, for which a set of possible reference or house-keeping genes is not available, Tseng et al. [8] suggested a method to select a set of non-differentially expressed genes specifically for the actual data set at hand. This method is an adaptation to two-channel arrays of the invariant difference selection algorithm (IDS) [18] proposed for the normalisation of single-channel oligonucleotide microarrays. A gene *g *is considered to be rank-invariant on an array, if the difference of the ranked Cy5 and Cy3 intensities is less than a threshold *d *and the average of the ranked intensities is not among the highest or lowest *l *ranks. For each array *j*, the set of rank-invariant genes $S_{j}^{I}$ is determined by the following expression:

(1) $$\begin{array}{l} S_{j}^{0}=\{g:|\text{r}({\text{Cy5}}_{jg})-\text{r}({\text{Cy3}}_{jg})|<d\\ \wedge l<(\text{r}({\text{Cy5}}_{jg})+\text{r}({\text{Cy3}}_{jg})/2<\left(G-l\right)\} \end{array}$$

where r(Cy5_*jg*_) and r(Cy3_*jg*_) are the ranks of the intensities and *G *is the number of spotted genes. To select a more conserved set of rank-invariant genes, Tseng et al. [8] proposed an iterative procedure of this method:

(2) $$\begin{array}{l} S_{j}^{i}=\{g:g\in S_{j}^{\left(i-1\right)}\\ \wedge |\text{r}({\text{Cy5}}_{jg})-\text{r}({\text{Cy3}}_{jg})|<p|S_{j0}^{i-1)}|\} \end{array}$$

where $S_{j}^{i}$ is the set of selected genes for array *j *in iteration *i*. The procedure starts with the starting set of genes $S_{j}^{\left(i-1\right)}=S_{j}^{0}$ from equation (1). The parameter *p *defines the proportion of genes selected in each iteration step. The procedure stops after iteration *I *defined by $|S_{j}^{I}|=|S_{j}^{\left(I-1\right)}$, i.e. when the number of rank-invariant genes between two iterations does not change. Tseng et al. [8] used the following parameter settings: *d *= *pG*, *l *= 25 and *p *= 0.02. The selected invariant genes are used in the weighted loess regression with the following weights.

(3) $$w_{jg}=\{\begin{array}{cc} 1 & \text{if}\;\;g\in S_{j}^{I}\\ 0 & \text{otherwise} \end{array}$$

### Modified rank-invariant selection algorithm (In-vMod)

In the rank-invariant selection procedure of Tseng et al. [8] (InvTseng), the selected set of rank-invariant proteins does not cover the complete intensity range due to the upper and lower limits on the average intensity ranks. Hence, also the loess curve fitted to the rank-invariant genes will not cover the entire intensity range. In order to be able to normalise all probes on the array, one can extrapolate the curve to the lower and upper intensity limits, e.g. by extending the loess curve beyond the upper/lower intensity limits. However, since loess regression is a locally fitting method, the extrapolated values will depend heavily on the small set of most extreme data points at the intensity limits and might not provide a good fit to values outside the selected range. To reduce the effect of over-fitting, Tseng et al. proposed to replace the local regression by linear fits through a subset of the most extreme data points. However, in our example applications both these extrapolation methods failed to fit the M-values appropriately for the lower and upper intensities. We solved this issue by simply omitting the upper and lower rank intensity thresholds in the initial selection iteration, i.e. equation (1) is replaced by

(4) $$S_{j}^{0}=\{g:|\text{r}({\text{Cy5}}_{jg})-\text{r}({\text{Cy3}}_{jg})|<pG\}$$

With the parameter settings proposed by Tseng et al. [8] the InvMod method applied to our data sets typically stopped after two to three selection iterations and selecting a varying number of approximately 10% to 20% of the proteins. To control the size of the set of rank-invariant proteins, we changed the stopping criterion of the procedure to $|S_{j}^{I}|\leq kG$, where *k *is the desired proportion of rank-invariant proteins in the selected set. In the simulation study and the evaluation of the real data set described in the next section we set *k *to 0.25, i.e. 25% of probes will be considered rank-invariant on each array. In addition, we set the proportion of selected proteins per iteration step to *p *= 0.99. This modification has a major impact on the procedure, since it greatly increases the number of iterations and leads to a selection algorithm that is more robust to outliers and differentially expressed proteins in situations in which the proportion of such proteins is large. Finally, we incorporated a weighting scheme, which takes the invariance of the probes regarding their rank differences into account. The proteins are weighted according to the standardised negative rank difference:

(5) $$w_{jg}=\{\begin{array}{ll} \frac{\max_{h\in S_{j}^{I}}(\Delta_{jh})-\Delta_{jg}}{\max_{h\in S_{j}^{I}}(\Delta_{jh})} & \text{if}\;\;g\in S_{j}^{I}\\ 0 & \text{otherwise} \end{array}$$

where Δ*_ig _*= |r (Cy5_*jg*_) - r (Cy3_*jg*_)| is the absolute difference of the ranked intensities of protein *g *in array *j*.

### Rank difference weighted global loess (RDWGL)

The weighting scheme described in equation (5) was applied to all probes on the array in order to perform a global loess normalisation with weighted probes. Here the term 'global loess' means that all probes are used in the loess fit (see [Additional file 1: R-implementations of the normalisation methods] for more details).

### Data processing and statistical analysis procedure

To correct for background effects we used the recommended normal-exponential convolution method 'normexp' [19,20] with an offset of 50 to stabilise the variances for probes with small intensity values. The 'normexp' method models the observed probe signal intensities as a mixture of a normal component representing the background noise and an exponential component representing the signal. Model parameters are estimated by maximum-likelihood estimation [20].

The background-corrected slides were within-array normalised by applying the modified loess normalisation methods described above. In addition, performances were compared with three standard within-array normalisation methods. The first of these methods is the well-known global loess normalisation (GL) which fits a non-linear loess curve where equal weight is assigned to all probes. The second method is the variance stabilising normalisation (VSN) of Huber et al. [21] that utilizes the arcsine transformation to stabilize the variance of the transformed intensities to be approximately independent of the mean intensities. Both methods assume most of the features on the arrays are not differentially expressed. Finally we included the generalized procrustes analysis (GPA) into our comparison. Procrustes analysis is a least-squares method for translation, rotation, scaling and aligning matrices that share the same dimension in order to maximize their agreement. The GPA normalisation is free of any statistical assumptions and thus according to the authors capable for the normalisation of boutique arrays [22]. All methods were also compared with nonnormalised data (NN), e.g. using the M-values of the background-corrected slides.

Individual slide effects are corrected for by A-quantile normalisation between arrays. This is done with data sets derived from all within-array normalisation methods except VSN, as the VSN method already incorporates a between-array normalisation procedure. A-quantile normalisation performs quantile-transformation of the A-values, so that the empirical distribution of the A-values is the same across all arrays.

To identify proteins that are differentially expressed between groups we used linear models and the empirical Bayes method by Smyth et al. (limma) [23]. The resulting raw p-values were used to reorder the list of proteins and to construct empirical ROC curves.

In the application on pancreatic cancer data multivariate classification rules were constructed for discriminating between different sample types. Multivariate classifiers were built by applying the nearest shrunken centroid classification method called PAM ('Prediction Analysis of Microarrays') [24], which selects from the full data set a subset of probes capable of discriminating between the classes based on their joint expression profiles. Optimal PAM threshold parameters were determined in an internal ten-fold cross-validation step, while the misclassification errors of the classifiers were estimated by an outer .632 bootstrap loop incorporating 100 bootstrap samples ([25,26]).

## Results and Discussion

### Simulation study

To compare within-arry normalisation procedures for boutique dual-colour antibody microarrays a simulation study was performed. The simulation was based on data generated by self-self incubations of plasma samples on twenty antibody microarrays in a dual-colour mode. The array layout and protocols are described in detail elsewhere [6,27]. In brief, the array comprises 1,800 data points representing 810 different antibodies in duplicates. The majority of target proteins was selected based on regulation in cancer-related transcriptional studies. In addition, positional controls, negative controls as well as a set of five potential housekeeping controls were integrated in replicates of 16 to 18 probes.

#### Simulation setup

In the simulation study we focused on situations in which the assumptions of the global loess normalisation are violated, i.e. in situations where a large proportion of proteins is differentially expressed or the distribution of up-and down-regulated proteins is asymmetrical. For each scenario 100 data sets were simulated by randomly as-signing a balanced number of arrays to a 'tumour' group and the remaining arrays to a 'control' group. Depending on the scenario, a fixed proportion of proteins were randomly drawn and set as being differentially up-or down-regulated. Then, for these probes a location shift was introduced to the M-values of the arrays defined as 'tumour' samples by addition or subtraction of the absolute values of random draws from the *N*(0.1, 0.1) distribution left-truncated at zero. This procedure leaves the mean intensity values (A-values) unchanged. After shifting the M-values, the modified dye intensities are Cy3* = 2^(*A *^^+*M/*2) ^and Cy5* = 2^(*A*-*M/*2) ^(see [Additional file 2: R-script to perform the simulation study] and [Additional file 3: RData-file containing the self-self hybridised dual-color microarray data set] for details about the simulation study).

#### Simulation results

**Figure 1:**
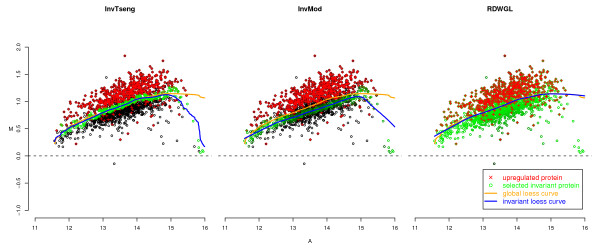
**Loess curves according to global loess (GL), rank-invariant loess (InvTseng), modified rank-invariant loess (InvMod) and rank difference weighted global loess (RDWGL) normalisation**. The selected rank-invariant proteins are shown in green. To illustrate the effect of a strong upregulation, the 40% of proteins simulated as being upregulated were generated by random draws from a *N*(0.3, 0.1) distribution left-truncated at zero and are shown in red.

**Figure 2:**
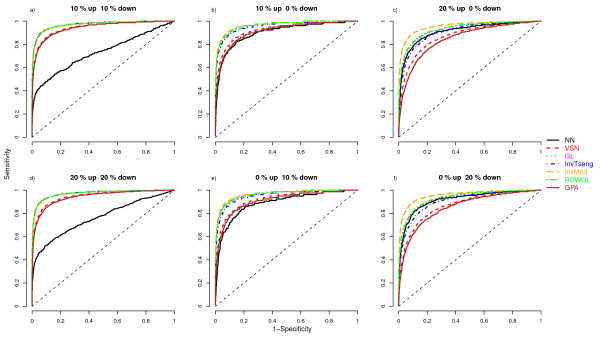
**ROC curves showing the median sensitivity and specificity across the 100 simulation steps for scenarios with a small proportion of differentially expressed proteins and symmetric differential expression**.

**Figure 3:**
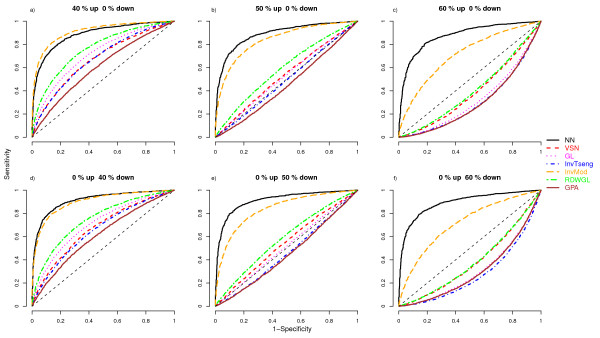
**ROC curves showing the median sensitivity and specificity across the 100 simulation steps for scenarios with a large proportion asymmetrical differential expression of proteins**.

**Figure 4:**
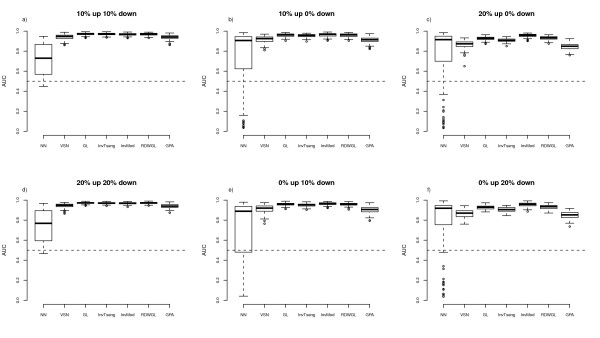
**Boxplot of the AUC-values for the ROC curves across the 100 simulation steps for scenarios with a small proportion of differentially expressed proteins and symmetric differential expression**.

**Figure 5:**
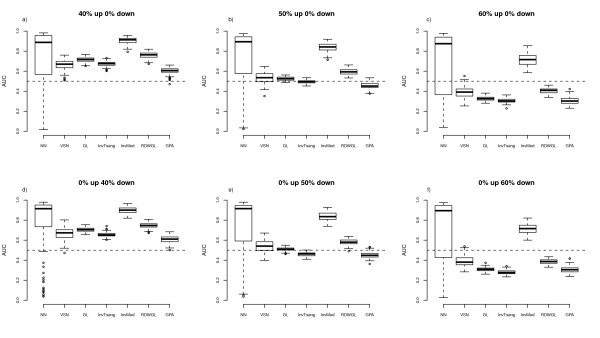
**Boxplot of the AUC-values for the ROC curves across the 100 simulation steps for scenarios with a large proportion asymmetrical differential expression of proteins**.

To assess the performances of the normalisation methods the respective MA-plots and loess curves were plotted for the different scenarios. The MA-plot and loess curves for one example simulation are displayed in Figure 1 for the rank-invariant selection algorithm (InvTseng), the modified rank-invariant procedure (InvMod) and the global loess using the rank difference weights (RDWGL) in combination with the unmodified global loess (GL). The global loess curve is clearly moved upwards by the large proportion of upregulated probes compared to the InvMod procedure and also to a lesser extend compared to InvTseng (Figure 1). The weights introduced in the RDWGL method do not result in a noticeable difference between the RDWGL and GL curves, indicating that the RDWGL curve was nearly as much affected by the upregulated probes as was the unmodified global loess curve. When the same proportion of probes is generated as being downregulated by the same amount, the resulting loess curves behave equivalently. To judge the impact of the tested normalisation procedures on the ability to correctly detect differentially expressed features in the various simulation scenarios, differential expression analysis was performed using the limma method. The respective results were summarized as empirical median ROC curves by estimating the median sensitivity and specificity values across all 100 simulation runs per scenario (Figure 2, 3). The related variability associated with the normalisation methods across the simulation runs was assessed by boxplots of the area under the ROC curves (Figure 4, 5). In situations were most of the proteins are not differentially expressed and when there is a symmetrical regulation of differential expressions in both directions, all normalisation methods perform well. This is the case for the first scenario (Figure 2a) where in total 20% of the proteins are differentially and symmetrically expressed. Even by increasing the proportion of differentially expressed proteins to 40% but keeping the expression symmetrical (Figure 2d), all normalisation methods still perform well. Also in cases where a small proportion of 10% of the proteins (Figure 2b, e) is asymmetrically and differentially expressed, all methods show a reasonable performance. Only VSN and the GPA normalisation exhibit worse performances than all other methods. This might be either due to differences in the within-array normalisation or due to the different between-array normalisation approach that is part of the VSN algorithm. In scenarios where the expression is more asymmetrical and therefore assumption (B) is violated, the performances of the normalisation methods start to deteriorate and the ROC curves show a decrease in sensitivity and specificity, in particular for the InvTseng method, but also for the global loess (GL) and RDWGL methods. Only the InvMod method is not much affected (Figure 2c, f, 3). Interestingly, the median ROC curves for the non-normalised simulated data sets (NN) show that on average across the 100 simulations, the performance of limma on non-normalised data is not much affected by an increase in the proportion of differentially expressed features or by an asymmetrical distribution of regulated features (Figure 3). However, the AUC-boxplots demonstrate that the ability to detect differentially regulated features by limma varies considerably across the 100 simulations (Figure 4, 5). That is, while on average the performance on non-normalised data can be quite reasonable, individual data sets can show poor sensitivities and specificities, resulting in AUC values near zero. In fact, the empirical 95% confidence intervals for non-normalised data encompass AUC = 0.5 for all scenarios, which means that the performances are not significantly better than a random assignment of features to the groups of either differentially or non-differentially expressed features. For increasing numbers of assymetrically regulated proteins, all normalisation methods fail to reliably detect simulated expression differences with the exception of the here introduced InvMod procedure (Figure 3a, b, d, e). Using this method median AUC levels of at least 0.7 could be obtained even for scenarios in which 50% of the proteins were asymmetrically up or down regulated (Figure 5a, b, d, e). Amongst the tested methods, the InvMod method is the only normalisation method that is robust against asymmetrically regulated proteins. This might be due to several modifications that were introduced to the original InvTseng method. For the InvTseng method, the upper and lower thresholds of the average ranked intensities used in the first selection iteration lead to a set of rank-invariant proteins that does not cover the complete intensity range and necessitates an additional extrapolation. This extrapolation often fails and thus the loess fit can not model proteins with extreme signal intensities. In addition, in each iteration a small proportion of proteins with low rank differences are selected. This usually leads to a fast convergence of the selection algorithm after a few iterations with highly variable numbers of selected proteins among the different arrays. In our simulation study, the set of proteins selected by the InvTseng method often consists of a large number of falsely selected differentially expressed proteins. Thus the resulting loess fit was often very similar to the GL fit, or even worse for situations with strong assymetrical regulation. The improved InvMod selection accounts for proteins with extreme intensities and thus does not necessitate additional extrapolation. In each selection iteration the algorithm selects a large proportion of proteins with a low rank difference. This leads to many iterations in which the rank differences are recalculated. Thus, the selection algorithm slowly selects a more consistent set of rank-invariant proteins. Furthermore, the algorithm stops once a desired proportion of proteins is selected. Thereby the number of rank-invariant proteins does not vary between arrays. Finally, the improved weighting scheme protects against falsely selected differentially expressed proteins. All these slight improvements of the original procedure of Tseng et al. [8] lead to a highly robust normalisation even in cases where the expression is expected to be asymmetrical and in which other normalisation methods fail. Note that the weighting scheme alone, as realised in the RDWGL method, can not fully catch the effect of a large number of asymmetrical regulated proteins, but in general leads to a loess fit that is only slightly more robust than the unmodified global loess method. In extreme situations where 60% of the proteins were asymmetrically regulated, the performance of the proposed In-vMod method decreases (Figure 3c, f). However, compared to all other normalisation methods investigated the InvMod method is still the most robust against asymmetrically regulated proteins showing AUC values of at least 0.6 (Figure 5c, f).

#### Data

In addition to the simulation study, we compared the performances of the different normalisation methods on a pancreatic cancer data set. The data were generated using the same antibody microarray platform as in the simulation study for measurements of protein abundance in urine samples. Six samples derived from patients suffering from pancreatic cancer and six samples derived from healthy controls were competitively incubated with a common reference in a dual-colour approach as described before [6]. Duplicate measurements lead to a total of 24 arrays. The slides were background-corrected and normalised as described above. A good normalisation method should remove unwanted technical bias in the data and thus improve the classification with respect to biological variation. Therefore, we assume that normalisation efficiency can be assessed by comparing the respective misclassification rates within a real data study (see [Additional file 4: R-script to perform the evaluation of the pancreatic cancer data set] and [Additional file 5: RData-file containing the pancreatic cancer data set] for details).

#### Results

**Figure 6:**
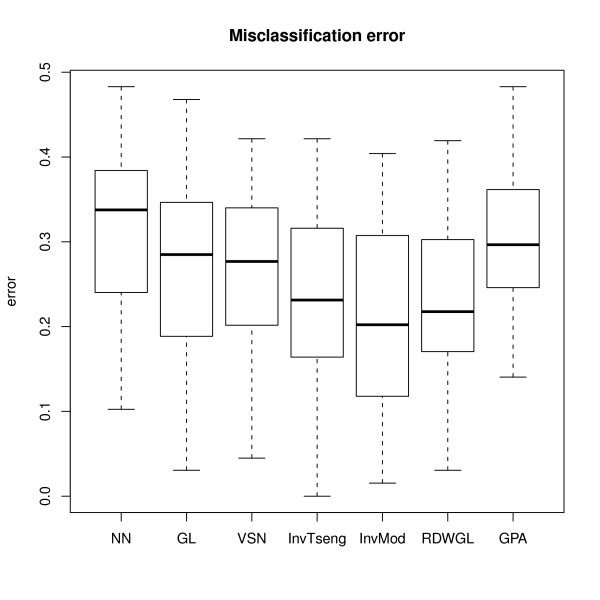
**Bootstrap estimated misclassification errors of the nearest shrunken centroid classifiers for the prediction of pancreatic cancer**.

The misclassification errors for the nearest shrunken centroid classifiers constructed for the pancreatic cancer data set were calculated for 100 bootstrap samples. Since the dataset comprises statistically dependent technical replicates, we expect the estimated misclassification errors to be optimistically biased. Still, a comparison of the performances of the different normalisation methods is possible, since the datasets are affected identically. The highest misclassification rates are observed for the non-normalised (NN) data (Figure 6). This indicates that normalisation is needed. A normalisation of the data by VSN, GPA or GL results in slightly lower misclassification errors than NN. The RDWGL, InvTseng and InvMod methods have the lowest misclassification errors. These three methods can at least partially account for possibly asymmetrically expressed proteins as observed in the simulation study (Figure 2, 3, 4, 5). Thus, it is likely that the reduction in error compared to global loess is at least partly due to asymmetry in regulation of the proteins measured by the boutique dual-colour antibody microarray. In-vMod exhibited the lowest misclassification error in this study, which is consistent with its clearly superior performance in the simulation study.

## Conclusions

We compared different within-array normalisation methods for the normalisation of boutique dual-colour antibody microarrays comprising several hundreds to some thousands of features. The focus was on situations where due to a likely selection bias the usual assumptions for most within-array normalisation methods are violated, i.e. the number of differentially regulated proteins is large and/or the regulation is asymmetrical. In these situations a global loess normalisation method, that is based on all features of the array, will artificially shrink the M-values to zero and thus possibly hide present differential expression. A possible solution is the use of control features which are known to be regulated in a constant manner independently of the experimental settings. However, in current protein arrays such control features are often missing or are underrepresented. In the case of boutique gene expression arrays titration series of a microarray transcript pool (MSP) constructed from transcript libraries have been proposed for building control features for normalisation [13,14]. For antibody arrays a similar approach might be the incorporation of a titration series of polyclonal polyspecific antibodies. In this manuscript we followed the alternative route, namely to base within-array normalisation on proteins that are not differentially regulated in the data set at hand. In order to find such probes, Tseng et al. [8] proposed an invariant selection algorithm which selects rank-invariant genes in dual-colour gene expression microarrays. We modified this algorithm in order to adapt it to the more challenging situation of dual-colour protein microarrays and compared it with the original as well as with other standard within-array normalisation methods. In a simulation study we demonstrated the outperformance of the established normalisation algorithm especially in situations where the usual assumptions for global normalisation methods are violated. Based on a real data set, the improved normalisation method lead to a superior classification of urine samples with respect to their actual disease state.

## Authors' contributions

MS conceived the improved invariant selection algorithm and was responsible for the simulation study and bioinformatic analysis of the pancreatic cancer data set. MS, CS, MZ, and AB were involved in the design of the simulation study. CS and JDH designed the antibody microarray, and performed the experiments. MS, CS and MZ prepared the manuscript, which was revised and approved by all authors.

## Supplementary Material

Additional file 1 **R-implementations of the normalisation methods**. The file contains R-functions for the different normalisation methods described in the article. Click here for file

Additional file 2 **R-script to perform the simulation study**. The file contains the R-script to perform the simulation study described in the article. The script is organized in three parts. The first part will generate semi-artificial data sets based on the self-self hybridised dual-color microarray data. The second part describes the simulation study and the third part will generate figures of the results, e.g. MA-plots, the ROC-plots and the boxplots of the AUC values. Click here for file

Additional file 3 **RData file containing the self-self hybridised dual-color microarray data set**. The file contains the self-self hybridised dual-color microarray data set used in the simulation study (see [Additional file 2: R-script to perform the simulation study]). Click here for file

Additional file 4 **R-script to perform the evaluation of the pancreatic cancer data set**. The file contains the R-script to perform the evaluation of the pancreatic cancer data set using prediction analysis of microarrays (PAM) and to generate boxplots of the bootstrap estimated misclassification errors. Click here for file

Additional file 5 **RData file containing the pancreatic cancer data set**. The file contains the pancreatic cancer data set (see [Additional file 4: R-script to perform the evaluation of the pancreatic cancer data set]). Click here for file
